# Early neural shift detection using functional magnetic resonance imaging: a pilot study with Parkinson’s disease patients undergoing istradefylline and hybrid assistive limb interventions

**DOI:** 10.3389/fneur.2026.1816153

**Published:** 2026-05-15

**Authors:** Ken-ichi Tabei, Keita Matsuura, Naoko Nakamura, Hiroyuki Kajikawa, Hidekazu Tomimoto, Akihiro Shindo

**Affiliations:** 1 Graduate School of Industrial Technology, Advanced Institute of Industrial Technology, Tokyo Metropolitan Public University Corporation, Tokyo, Japan; 2 Department of Neurology, Mie University, Tsu, Mie, Japan; 3 Department of Neurology, Suzuka Kaisei Hospital, Suzuka, Mie, Japan

**Keywords:** early neural shift, fMRI, hybrid assisted limb, istradefylline, Parkinson’s disease

## Abstract

**Background:**

Parkinson’s disease (PD) presents with not only motor symptoms but also non-motor features, including cognitive impairment. Although conventional assessments usually require extended observation periods to detect functional changes, more sensitive measures may enable the detection of changes over shorter intervals. Istradefylline, an adenosine A2A receptor antagonist, and the Hybrid Assistive Limb (HAL) device are emerging therapeutic options for PD. However, their short-term functional effects remain unclear, especially when assessed using traditional clinical and neuropsychological tools.

**Methods:**

We enrolled six PD patients (Hoehn and Yahr stages II–III) in our prospective, single-center study. Three patients received istradefylline for 1 month in addition to stable antiparkinsonian medication, and three underwent a 2-week HAL-assisted rehabilitation program while continuing their usual medication. Functional magnetic resonance imaging (fMRI) scans and a battery of clinical, cognitive, mood, sleep, and quality of life assessments were conducted at baseline and follow-up (2 weeks for the HAL-assisted rehabilitation group and 1 month for the istradefylline group). We compared the changes in task-related fMRI activation and traditional clinical endpoints.

**Results:**

All six patients completed the study without serious adverse events. After 1 month of istradefylline treatment, fMRI findings showed increased activation in multiple brain regions associated with motor and cognitive control. In contrast, clinical outcome measures (including cognitive, motor, mood, sleep, and quality-of-life scores) showed no significant improvement at follow-up in either the istradefylline group or the HAL-assisted rehabilitation group.

**Conclusion:**

Short-term istradefylline administration elicited measurable changes in brain activation patterns without corresponding clinical or cognitive gains, which suggests that fMRI may serve as a more sensitive biomarker of early, subthreshold neural adaptations than conventional assessments. However, these findings must be interpreted with caution due to the small sample size and exploratory nature of the study.

## 1 Introduction

Parkinson’s disease (PD) is a progressive neurodegenerative disorder characterized by not only motor symptoms but also a range of non-motor manifestations, including cognitive impairment (1). The clinical and societal burden of PD is expanding rapidly worldwide, necessitating the development of more effective and sensitive evaluation strategies for emerging treatments (2). Because of the generally insidious course of PD progression, robust evaluation of treatment efficacy or disease modification typically relies on prolonged observation periods of several months to years. However, there has recently been growing interest in more multifaceted therapeutic strategies that combine pharmacological and non-pharmacological interventions (3). Such approaches raise the possibility that even relatively short treatment durations—in the order of several weeks—may induce measurable changes in neurological function.

Istradefylline, an adenosine A2A receptor antagonist, is an emerging class of pharmacotherapy for such combined therapies. By modulating both the dopaminergic and non-dopaminergic pathways, istradefylline reduces the “off” time in patients with PD, offering a potential avenue for more rapid functional improvement (4). Simultaneously, the use of a Hybrid Assistive Limb (HAL), a wearable robotic device, represents a non-pharmacological intervention aimed at improving motor function, gait, and overall quality of life in individuals with PD (5, 6). Although promising, the efficacy of these interventions has predominantly been assessed using conventional neuropsychological assessments and clinical rating scales, which may fail to detect subtle cognitive and functional changes over shorter durations because they often lack sufficient sensitivity for capturing early-stage or low-amplitude improvements (7).

In contrast, functional magnetic resonance imaging (fMRI) non-invasively assesses dynamic changes in brain activity and connectivity, which may serve as a more sensitive biomarker of early or subclinical improvements in PD. Previous research has shown that fMRI can detect subtle alterations in neural function that precede measurable deficits on neuropsychological tests in patients with PD (8, 9) and other neurodegenerative conditions (10, 11). Therefore, fMRI may provide an effective means to evaluate the short-term impact of pharmacological interventions, such as istradefylline, and HAL devices, by capturing brain-based changes that emerge well before conventional clinical measures can register meaningful improvement.

Previous research on the clinical efficacy and disease progression of therapeutic interventions for PD has typically investigated effects spanning several months to years. However, recent evidence has suggested that certain neurobiological and functional changes are detectable over considerably shorter intervals. Therefore, we aimed to investigate whether a 2-week to 1-month intervention period can induce early, subthreshold alterations in brain function that precede measurable improvements on standard clinical scales. We structured the study as a pilot trial (i.e., by enrolling a small cohort of participants) to explore the feasibility of short-term intervention assessments using fMRI activity as a potential early biomarker. Our findings will inform the design of future large-scale or long-term clinical trials to further validate and extend our results.

## 2 Materials and methods

This study was conducted as a pilot trial to investigate the feasibility and preliminary impact of short-term interventions on both clinical outcomes and task-based fMRI findings in individuals with PD. Specifically, participants receiving istradefylline were evaluated 1 month after treatment initiation, whereas those undergoing HAL-assisted rehabilitation were assessed 2 weeks after commencing therapy. These time points were chosen to determine whether functional or neurobiological changes can be captured earlier than more conventional observation periods used in PD research (which often span several months). Given our exploratory approach, we used a small sample size with the aim of assessing practical considerations (e.g., participant adherence and magnetic resonance imaging [MRI] protocol tolerability).

### 2.1 Participants and ethics

Six participants were recruited from the Department of Neurology, Mie University Hospital. Inclusion criteria were: a diagnosis of PD according to the Movement Disorder Society Clinical Diagnostic Criteria (12), Hoehn and Yahr stage II–III, stable antiparkinsonian medication regimen for at least 4 weeks prior to enrollment, and capacity to provide informed consent. Exclusion criteria were: contraindications to MRI scanning, severe comorbid neurological or psychiatric disorder, and severe cognitive impairment that restricted full understanding of study procedures. The study protocol was approved by the Institutional Review Board of Mie University Hospital and Suzuka Kaisei Hospital (approval numbers H2019-134 and 2016–02, respectively). All participants provided written informed consent in accordance with the Declaration of Helsinki.

### 2.2 Study design and interventions

This was a prospective single-center study designed to evaluate short-term changes in brain function following a 2- to 4-week intervention period. Three participants were assigned to take istradefylline once every morning (20 mg/day) in addition to their stable antiparkinsonian medication regimen. Another three participants underwent a 9-day HAL-assisted rehabilitation program, which comprised 60-min lower-limb training sessions. Baseline assessments were conducted before the intervention, and follow-up evaluations were performed 2 weeks after the start of the HAL-assisted rehabilitation program and 1 month after the initiation of istradefylline administration. It is important to note that the differing intervention durations (1 month for istradefylline and 2 weeks for HAL) were chosen to reflect the earliest practical and clinically relevant assessment points for each respective modality. The objective of this study was not to directly compare the efficacy of the two modalities, but rather to evaluate whether fMRI could capture early neural shifts at these distinct early follow-up intervals.

### 2.3 Clinical and neuropsychological assessments

To comprehensively evaluate the cognitive, motor, mood, and quality-of-life changes associated with the interventions, we used a range of validated clinical and neuropsychological instruments. Cognitive function was assessed using the Mini-Mental State Examination (13), a brief and widely used measure to evaluate global cognition, and the Frontal Assessment Battery (14), which is sensitive to detecting executive dysfunction commonly observed in patients with PD. Additionally, we administered Raven’s Colored Progressive Matrices (15) to evaluate nonverbal reasoning and the Benton Visual Retention Test (16) to assess visuospatial memory. The Trail Making Test A (17) was used to measure processing speed and attention, and the Kohs Block Design Test (18) assessed visuo-constructive abilities. Figure copying tasks, such as copying five types of figures (i.e., vertical diamond, two-dimensional cross, three-dimensional block, three-dimensional pipe, and triangle within a triangle) (19), and the Necker cube test (20) were administered to further evaluate visual perception, spatial cognition, and executive control processes. Motor function was evaluated using the Unified Parkinson’s Disease Rating Scale (21), which is an established clinical metric of PD motor dysfunction severity. This was supplemented by the Freezing of Gait Questionnaire (22) to quantify gait disturbances. The Timed Up and Go test (23) was used to measure functional mobility and balance. Mood and apathy were assessed using the Apathy Scale (24) and the Center for Epidemiologic Studies Depression Scale (25), to detect changes in motivation and depressive symptoms that accompany treatment. Sleep quality was evaluated using the Epworth Sleepiness Scale (26) and the Parkinson’s Disease Sleep Scale-2 (27) because sleep disturbances are a critical non-motor symptom of PD. Finally, we assessed quality of life using the Parkinson’s Disease Questionnaire (28), which encompasses the physical, emotional, and social domains relevant to daily functioning. All assessments were conducted at baseline and the follow-up time points for each intervention. The behavioral, clinical, and cognitive data were compared with the fMRI-based measurements to determine whether fMRI offers a more sensitive and timely index of short-term intervention effects than the traditional neuropsychological and clinical scales.

### 2.4 FMRI data acquisition

MRI data were acquired on a 3.0-tesla scanner (Siemens Magnetom Verio) using a T2*-weighted gradient-echo echo-planar imaging sequence (repetition time: 3490 ms; echo time: 35 ms; flip angle: 90°; matrix size: 96 × 96; field of view: 240 mm, and 30 5-mm thickness slices with no gap). Anatomical reference MRI data were acquired using a T1-weighted turbo field-echo imaging sequence (repetition time: 5900 ms; echo time: 2.63 ms; flip angle: 15°; field of view: 224 × 224 mm, and slice thickness: 1 mm).

### 2.5 FMRI preprocessing and analysis

Data were analyzed using SPM12 (Wellcome Trust Center for Imaging, London, United Kingdom) running on Matlab (MathWorks Inc., Natick, MA). Each scan was realigned to the first image of each scanning condition to correct for movement artifacts. The realigned data were spatially normalized to the Montreal Neurological Institute template and resliced at 3 × 3 × 3 mm. The resulting images were smoothed with an 8-mm (full width at half maximum) Gaussian filter. Individual statistical maps (fixed effect) were calculated for the foot and go tasks (12) and changes from baseline to follow-up (main effect). The onsets were defined as the onset time of the stimulus. For each participant, we computed contrasts for post-foot task > pre-foot task and post-go task > pre-go task. During the fMRI scanning, participants performed a foot-tapping task and a Go/No-Go task using a block design. For the foot-tapping task, the protocol consisted of 3 blocks of active movement (lasting 60 s) alternating with 3 blocks of rest (lasting 60 s). For the Go/No-Go task, stimuli were presented for 60 s with an inter-stimulus interval of 1 s, alternating with 3 Go/No-Go task trials and 3 blocks of rest trials per block. Behavioral performance, including accuracy and reaction times, was recorded during the scan to monitor task compliance. The statistical threshold used to report group activations was set at *p* < 0.05, corrected for the whole brain (false discovery rate). The Montreal Neurological Institute coordinates of the significant voxels with the highest t-values were recorded and assigned to anatomical regions.

### 2.6 Statistical analysis of clinical and cognitive data

Changes in motor and cognitive measures were compared between pre- and post-intervention (i.e., baseline and 2 weeks or 1 month). Due to the extremely small sample size (*n* = 3 per group, *N* = 6), strict non-parametric statistical methods were employed to avoid violating assumptions of normality. Specifically, the Wilcoxon signed-rank test was utilized for all within-group pre- and post-intervention comparisons of clinical and neuropsychological measures. Because of the limited statistical power, between-group statistical testing was not performed. The fMRI activation maps were similarly analyzed focusing on within-group contrasts (post- vs. pre-intervention). Statistical analyses were conducted using IBM SPSS Statistics software version 27 (IBM Corp., Armonk, NY).

### 2.7 Endpoints and outcome measures

The primary endpoint was the change in task-related activation patterns derived from fMRI during the short intervention period. Secondary endpoints were changes in neuropsychological test and motor rating scale scores. These measures were compared to evaluate the sensitivity of fMRI relative to standard clinical and cognitive assessments in detecting early therapeutic effects.

## 3 Results

### 3.1 Participant characteristics and data quality

A total of six patients with PD completed all study assessments (mean age: 77.8 ± 3.8 years; five males and one female). All six patients with PD completed the study with high compliance (>90%) and no serious adverse events. The three patients who received istradefylline adhered to the drug regimen.

### 3.2 FMRI analyses

**Figure 1:**
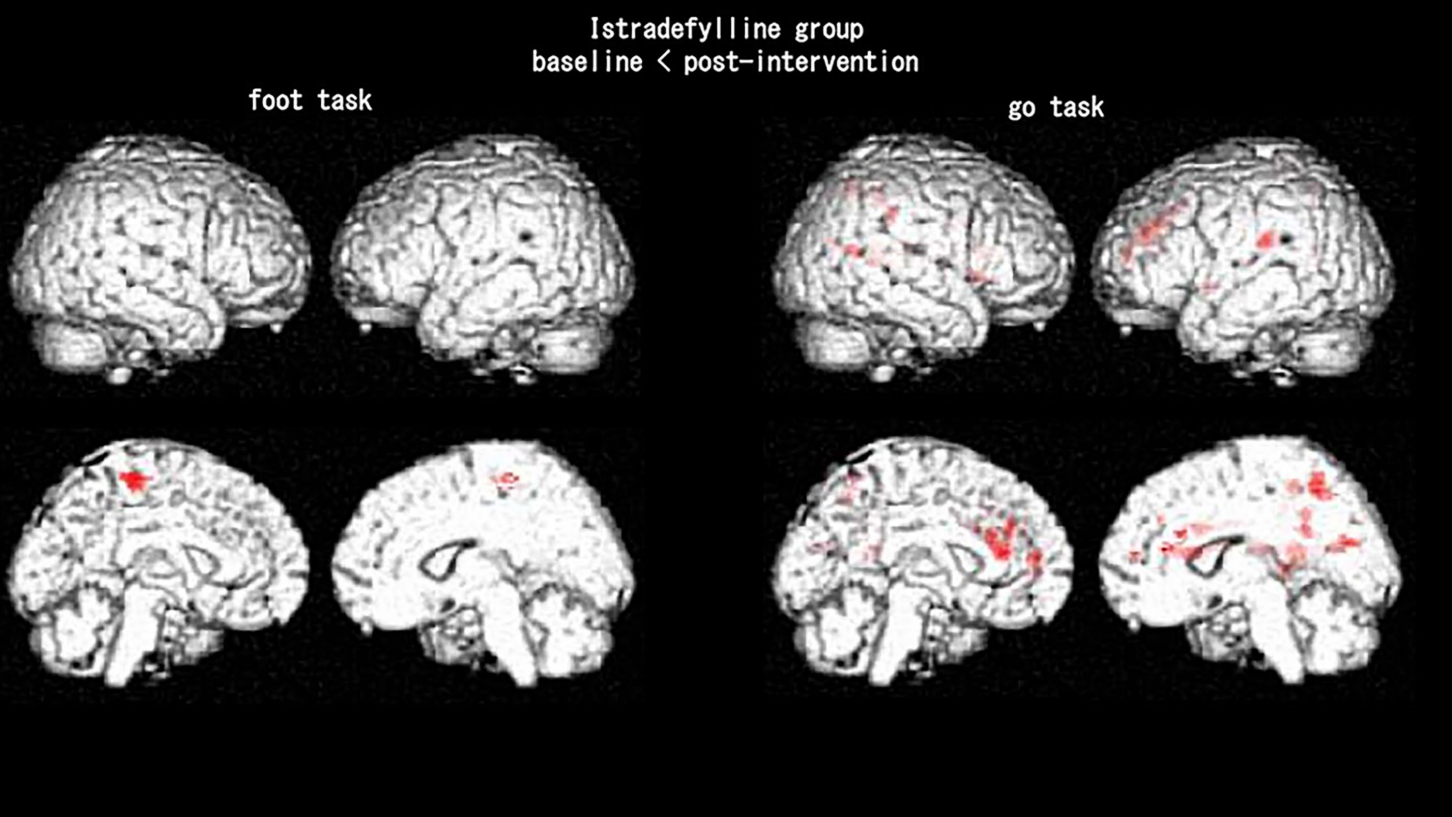
Brain activation changes in the istradefylline group after 1 month of treatment. Areas in red show increased activation post-intervention compared with baseline (baseline < post-intervention). No significant activation changes were observed in the hybrid assistive limb group.

The istradefylline group showed increased activation in the left paracentral lobule and left medial frontal gyrus during the foot task following 1 month of treatment (Figure 1). During the go task, the istradefylline group showed increased activation in the left postcentral gyrus, right precuneus, right caudate, right inferior parietal lobule, right posterior cingulate, right cingulate gyrus, right superior parietal lobule, left sub-gyral, left postcentral gyrus, and right precentral gyrus following 1 month of treatment (Figure 1). Patients who used the HAL device showed no significant brain activation changes following the intervention (Tables 1, 2).

**Table 1:** Clinical and neuropsychological assessment scores for the istradefylline group.

Assessments	Baseline		Post-intervention		*p*-value
	Mean	SD	Mean	SD	
Mini-Mental State Examination	19.3	5.5	21.3	4.0	0.157
Frontal Assessment Battery	7.7	3.5	8.3	4.5	0.317
Kohs Block Design Test	15.5	5.0	11.5	0.7	0.180
Raven’s Colored Progressive Matrices	19.0	2.8	15.5	3.5	0.180
Benton Visual Retention Test	2.5	0.7	2.5	0.7	1.000
Trail Making Test A	220.5	34.6	318.0	137.2	0.180
Center for Epidemiologic Studies Depression Scale	13.7	9.7	20.7	13.7	0.109
Apathy Scale	25.0	11.5	21.7	8.1	0.180
Figure Copying	8.0	5.6	7.3	6.7	0.317
Necker cube	1.0	1.0	0.7	0.6	0.317
UPDRS Part I	5.3	4.2	4.0	1.7	0.655
UPDRS Part II	15.7	9.0	15.7	9.3	1
UPDRS Part III	37.3	24.1	35.3	21.4	0.285
UPDRS Part IV	1.7	0.6	1.3	0.6	0.317
UPDRS total score	60.0	36.9	56.3	31.8	0.285
Parkinson’s Disease Questionnaire	145.3	73.3	106.7	12.7	0.180
Freezing of Gait Questionnaire	11.3	6.1	12.7	6.0	0.593
Epworth Sleepiness Scale	2.7	2.1	5.3	3.2	0.102
Parkinson’s Disease Sleep Scale-2	18.7	3.8	17.7	7.2	0.785
Timed Up and Go test	22.7	0.7	20.1	2.8	0.180

SD, standard deviation; UPDRS, Unified Parkinson’s Disease Rating Scale.

**Table 2:** Clinical and neuropsychological assessment scores for the hybrid assistive limb intervention group.

Assessments	Baseline		Post-intervention		*p*-value
	Mean	SD	Mean	SD	
Mini-Mental State Examination	18.3	5.0	20.0	4.6	0.180
Frontal Assessment Battery	9.3	5.8	9.7	5.0	1.000
Kohs Block Design Test	25.7	20.8	27.0	17.3	1.000
Raven’s Colored Progressive Matrices	23.5	9.2	25.3	5.1	0.180
Benton Visual Retention Test_	2.3	2.1	2.3	2.1	1.000
Trail Making Test A	326.5	44.5	318.0	56.6	0.317
Center for Epidemiologic Studies Depression Scale	14.0	12.2	15.0	11.4	0.180
Apathy Scale	19.7	7.6	20.7	2.5	1.000
Figure Copying	11.3	3.2	12.0	3.0	0.655
Necker cube	2.3	0.6	2.7	0.6	0.317
UPDRS Part I	3.7	3.5	4.0	3.6	0.317
UPDRS Part II	16.3	7.4	17.0	7.9	0.157
UPDRS Part III	26.7	10.3	24.3	12.1	0.102
UPDRS Part IV	3.0	2.6	3.3	2.3	0.317
UPDRS total	49.7	20.2	48.3	21.5	0.655
Parkinson’s Disease Questionnaire	100.3	9.5	102.0	14.9	0.655
Freezing of Gait Questionnaire	12.0	7.2	8.3	5.7	0.180
Epworth Sleepiness Scale	8.3	3.2	5.3	2.1	0.317
Parkinson’s Disease Sleep Scale-2	17.3	13.6	14.7	13.1	0.180
Timed Up and Go test	34.9	31.7	31.5	26.0	0.285

SD, standard deviation; UPDRS, Unified Parkinson’s Disease Rating Scale.

### 3.3 Clinical and neuropsychological outcomes

No significant changes were observed for any neuropsychological or clinical assessment scores in either the HAL-assisted rehabilitation or istradefylline group at 2 weeks and 1 month, respectively (Tables 1, 2).

## 4 Discussion

This study sought to determine whether fMRI can serve as a more sensitive biomarker of short-term therapeutic effects in PD patients than conventional clinical and neuropsychological assessments. Our findings indicated that a 1-month administration of istradefylline, an adenosine A2A receptor antagonist, increased activation in several brain regions associated with motor and cognitive control. These neural changes occurred despite the absence of significant improvement in clinical measures, cognitive measures, mood, sleep quality, or quality of life.

The discrepancy between robust fMRI signal alterations and the lack of corresponding clinical or cognitive gains is noteworthy. Previous research has suggested that fMRI can detect subtle, subthreshold changes in brain function before they manifest in behavioral or cognitive performance (10, 11). This is consistent with our results. Indeed, short-term interventions may trigger early neurobiological adjustments that are not immediately reflected in conventional clinical assessments. Although functional changes detected via fMRI may presage future behavioral improvements, our short observation period may have been insufficient for these neural adaptations to translate into measurable clinical benefits. Nevertheless, despite the short intervention periods (2 weeks for HAL-assisted rehabilitation and 1 month for istradefylline), our findings suggest that fMRI metrics are sensitive enough to detect early functional neural changes before clinical improvements become apparent.

This pilot study has several critical limitations that must be acknowledged. First and foremost is the extremely small sample size (*N* = 6; *n* = 3 per group). This severely limits statistical power, precludes robust between-group comparisons, and significantly increases the risk of both false-positive and false-negative findings. Consequently, the results must be interpreted as strictly exploratory. Second, the study design lacks a placebo or sham-rehabilitation control group. Without such controls, it is impossible to definitively attribute the observed fMRI changes specifically to the istradefylline or HAL interventions, as these neural shifts could also arise from practice effects (task repetition), spontaneous disease fluctuations, or scanner variability. Third, the differing intervention durations (1 month vs. 2 weeks) limit the ability to draw parallel conclusions about short-term effects across modalities. Institutional ethical regulations prevented the disclosure of individual-level data in this small cohort; future studies with larger sample sizes are needed to explore individual heterogeneity. Future large-scale, randomized controlled trials incorporating appropriate placebo/sham groups, where data can be safely anonymized, are essential to validate these preliminary findings and isolate the true treatment-related neural adaptations.

In conclusion, our preliminary findings suggest that fMRI may have the potential to detect early, subclinical neural adaptations before overt changes in conventional clinical scores emerge. However, this interpretation must be made with caution. The observed increases in brain activation do not necessarily indicate definitive therapeutic improvement; they could alternatively reflect compensatory neural mechanisms, task-related variability, or practice effects. Longitudinal studies are required to determine whether these short-term, early neural shifts eventually translate into sustained clinical benefits.

## Data Availability

The original contributions presented in the study are included in the article/supplementary material, further inquiries can be directed to the corresponding author.
